# Plastiglomerates from uncontrolled burning of plastic waste on Indonesian beaches contain high contents of organic pollutants

**DOI:** 10.1038/s41598-023-37594-z

**Published:** 2023-06-27

**Authors:** Dwi Amanda Utami, Lars Reuning, Lorenz Schwark, Gernot Friedrichs, Ludwig Dittmer, Ayu Utami Nurhidayati, Ahmad Al Fauzan, Sri Yudawati Cahyarini

**Affiliations:** 1 Research Center for Climate and Atmosphere, National Research and Innovation Agency Republic of Indonesia, Jl. Cisitu Sangkuriang, Bandung, 40135 Indonesia; 2 Institute of Geosciences, Kiel University, Ludewig-Meyn-Str. 10, 24118 Kiel, Germany; 3 Institute of Physical Chemistry, Kiel University, Max-Eyth-Str. 1, 24118 Kiel, Germany; 4 Oceanography Study Program, Institut Teknologi Bandung, Jl. Ganesha No. 10, Bandung, 40132 Indonesia

**Keywords:** Environmental sciences, Planetary science

## Abstract

This study reports on plastiglomerate and other new forms of plastic pollution in the tropical marine continent of Indonesia. Twenty-five samples were collected from an island beach in the Java Sea where plastiglomerate, plasticrusts, and pyroplastic were formed by the uncontrolled burning of plastic waste. The most common plastic types were polyethylene and polypropylene (PE/PP), as shown by ATR-FTIR spectroscopy. However, acrylates/polyurethane/varnish (PU) and a copolymer of styrene and acrylonitrile were found as well. This suggests that plastiglomerates can form from a wider variety of plastic polymers than previously reported. FTIR analysis also indicates thermo-oxidative weathering, making the charred plastic more brittle and susceptible to microplastic formation. A subset of the samples was analyzed for associated chemical contaminants. One plastiglomerate with a PU matrix showed high concentrations of phthalates. All samples had high concentrations of polycyclic aromatic hydrocarbons (PAHs), likely due to the burning of the plastic in open fires. The burning leads to a change in the physical and chemical properties of the plastics contained in the plastiglomerates. Plastiglomerate and plastic waste of similar origin are therefore often more weathered and contaminated with organic pollutants than their parent polymers. The highest PAH concentration was found in a plastitar sample. Plastitar is defined as an agglomerate of tar and plastics that adheres to coastal rocks. In contrast, our study documents a more mobile, clastic plastitar type. This clastic plastitar could pose an additional ecological risk because of its mobility. These new types of plastic pollution could be an important vector for chemical contamination of nearby coastal habitats such as coral reefs, seagrass meadows, and mangroves.

## Introduction

The accumulation of plastic waste has become a global problem since the beginning of mass production of plastic in the 1950s. Thirty-three billion tons of plastic are expected to be produced worldwide by 2050^1^. A significant portion of this ends up in the oceans, primarily due to mismanagement^2^. Plastic waste is the most important fraction of marine litter and persists in the environment due to its longevity and resistance to weathering and abrasion. The major impacts of plastic debris in the marine environment include entanglement or entrapment and ingestion by marine fauna^3–5^. In addition, marine plastic debris can be a vector for toxic persistent organic pollutants (POPs) via being ingested by invertebrates and transported to higher trophic levels, increasing the potential for bioaccumulation^6–8^.

Recently, new types of plastic formation with potential additional environmental impacts and chemical hazards have been introduced, known as plastiglomerate^9^. Plastiglomerate was initially described as a mixture of molten plastic with natural and artificial debris, divided into clastic and in situ types^9^. The definition of plastiglomerate has since been expanded beyond its original classification. Clastic plastiglomerate is described as scattered loose sediment lying on the surface of a beach, often near the vegetation line or buried beneath the sand^10^. In situ plastiglomerate is formed by molten plastic filling the vesicles and fractures of rocks^10^. Pyroplastic and plasticrusts are subtypes of plastiglomerate^10^. Their source is mainly attributed to the informal burning of plastic waste and campfires^10,11^. Pyroplastics are melted or burned plastics that have lost their original shape and color and sometimes resemble natural rocks^11^. Pyroplastic is usually formed from plastics without significant additional material, so its bulk density is often low enough to be buoyant in seawater^11^. Due to their buoyancy and durability, pyroplastics are discussed as a significant vector for alien invasive species^12^. The term plasticrust was first introduced for pieces of plastic encrusting the surface of rocks^13^. These plasticrusts were interpreted to form by scouring ropes and other plastic items on rocks in the intertidal zone^13,14^. Later the term was also used to describe crusts of molten plastic partly covering boulders and larger rocks on a beach^15^, very similar to the in situ plastiglomerate described by Corcoran et al.^9^. The agglutination of molten plastic with other natural components, such as rocks, increases their bulk density and limits their ability to be transported by wind or currents. This increases their potential to be buried and preserved, eventually becoming part of the Anthropocene record^9^. Another new plastic pollution type called plastitar does not conform to the previous classification. Plastitar is formed mainly from plastics and tar but may contain natural and artificial materials^16^. The observed plastitar was attached and immobilized on the surface of nearshore marine rocks^16^.

Plastiglomerates were first discovered on the beach of Hawaii^9^ and later studied in other parts of the world, such as Peru, the Canary Islands, England, Japan and Madeira (northeast Atlantic). Their polymers were mainly identified as polyethylene (PE), polypropylene (PP), and polyethylene terephthalate (PET)^9,11,13,15–18^, and they are mainly derived from packaging materials and marine ropes^9,13,15,18^. Exposure to sunlight and wave action can lead to their disintegration into smaller plastic pieces that may enter the marine food web, with unknown consequences for marine biota^11,12,15^. Thus, they pose a new challenge to the global plastic pollution problem. It has been postulated that this new plastic formation could be a vector for persistent organic pollutants released by the plastic-burning process^9,15,19^. On tropical beaches, this would add to the anthropogenic stress on tropical coastal ecosystems such as coral reefs, seagrass meadows, and mangroves ^20–25^, which are already under pressure from other harmful anthropogenic impacts. However, data on organic chemical contaminants in plastiglomerate and other similar forms of plastic pollution are hitherto missing.

In this work, we analyze for the first time the abundance and polymer type of plastiglomerate, pyroplastics, plasticrust, and plastitar from a part of the tropical marine continent of Indonesia. Presence of plastic waste can increase disease susceptibility in reef-building corals^26^, which may have negative consequences for reef-associated organism and people^27^. It is shown that this new type of plastic pollution is associated with persistent organic pollutants that could threaten the nearby already degraded coral reef system. A better understanding of this new type of plastic debris specific characteristics will therefore help guiding the worldwide marine conservation policy to protect tropical coastal ecosystems.

## Methods

### Study area and oceanographic setting

**Figure 1:**
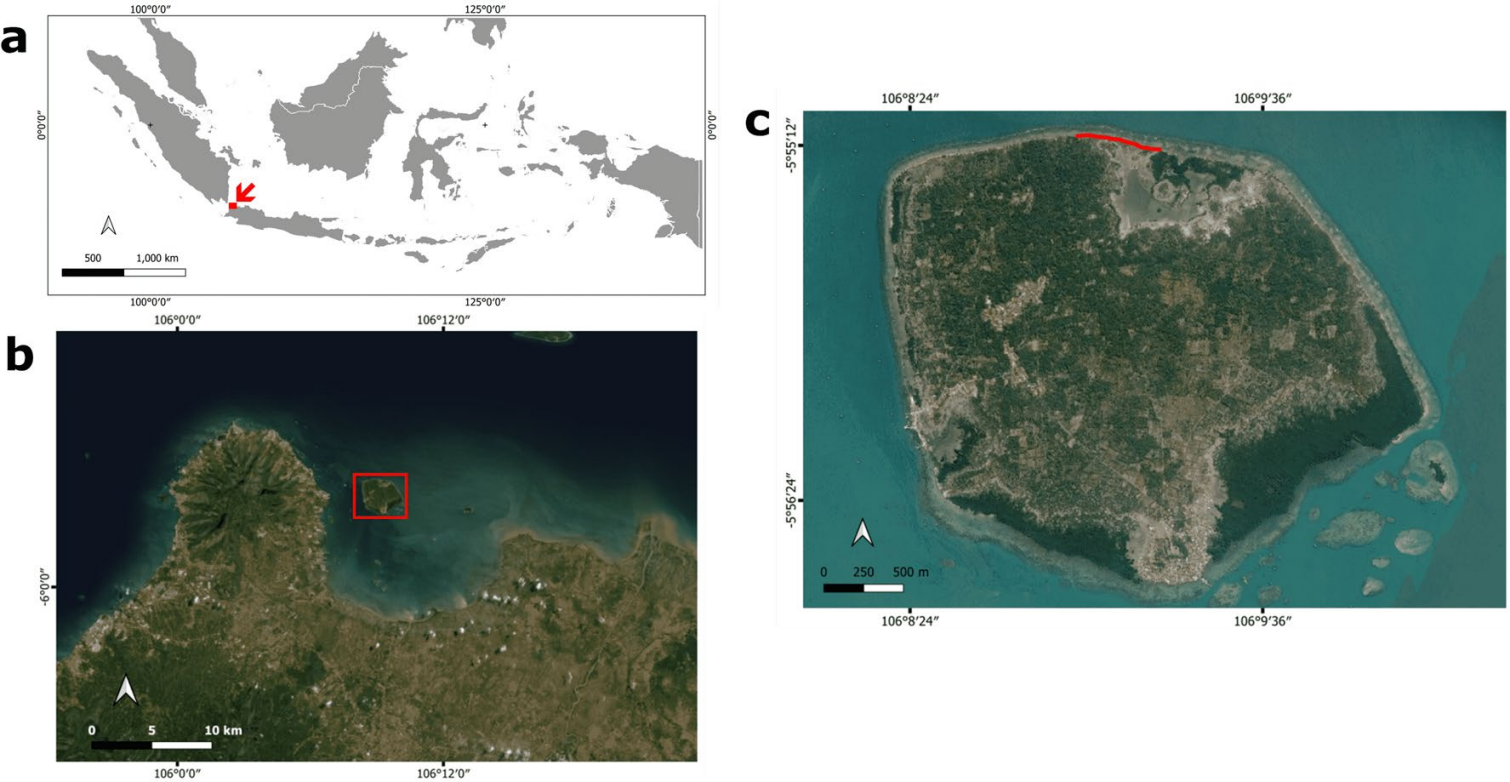
(**a**) The study area is located in the Java Sea, Indonesia, as indicated by the red arrow. (**b**) Panjang Island (red square) is located in the north of Banten Bay. Note the turbidity of the water in Banten Bay due to sediment load from the rivers. (**c**) Sampling site on the northern shore of Panjang Island (red line). Satellite image copyright: ESRI Satellite using QGIS ver.3.16.9.

The northern coast of Panjang Island in the Java Sea, Indonesia, was selected to investigate the presence of plastiglomerates and similar types of plastic pollution (Fig. 1). The site was chosen because it is adjacent to the three typical tropical coastal habitats: Coral reefs, seagrass meadows and mangroves. The beach is separated from a fringing reef by a shallow lagoon about 60 m wide with abundant seagrass meadows. The coral reefs in the northern part of Panjang Island are in moderate condition and consist of branching, tabular and massive corals^27^. Along the beach, the substrate is mainly rubble from branching corals. Towards the east, the beach transitions to a muddier substrate covered by a mangrove ecosystem (Fig. 1). Any plastic pollution present at the beach therefore could potentially contaminate all three tropical coastal ecosystems.

Panjang Island is located in Banten Bay, a shallow, semi-enclosed body of water generally 2–20 m deep. There are twelve islands in Banten Bay, of which Panjang Island is the largest (8.2 km^2^) and has more than 4000 inhabitants. Seven rivers flow into Banten Bay and contribute sand and mud to the seabed^28^. Seagrass, mangrove, and coral reef ecosystems dominate the coastal area. The most extensive seagrass cover is found around Panjang Island^29^. Mangroves are typical for beaches with muddy substrates^28^. There are coral reefs on Panjang Island's west, north, and east coasts, but their condition is relatively poor^28^. The beaches in Banten Bay are composed of coral debris and shell fragments of marine biota^28^, similar to other beaches in the Java Sea close to coral reef ecosystems^30,31^.

The monsoon climate influences Banten Bay in the Java Sea. Around Panjang Island, current velocities were measured between 0–2 and 0–2.4 km/h during the east and west monsoons, respectively. These weak ocean currents are responsible for sediment transport in the semi-enclosed Banten Bay^28^. Salinity in Banten Bay ranges between 29 and 32 ‰ and sea surface temperatures are between 28 and 30 °C. Tides in Banten Bay are predominantly semidiurnal and microtidal, with a mean tidal range of 0.50 m^32^.

Studies of marine debris in Banten Bay have found that plastic debris is a significant problem^33,34^. The movement of marine debris depends on the hydrodynamics within the semi-enclosed bay^34^. Plastic items such as PET bottles, plastic packagings such as cups and lids, and microplastics are common^33,34^. Model simulations indicate that 41% of the Banten Bay area will be covered with plastic litter by 2028 if no immediate action is taken^34^.

### Sampling

Samples were collected from a wave-exposed beach facing the open sea. The study site is located in a remote coastal area approximately 1 km north of the nearest village and is characterized by limited fishing activity. Three parallel, alongshore transects were surveyed to cover the entire beach from the shoreline to the vegetation line, including the upper intertidal and supratidal zones. Sampling was conducted at high tide. The three alongshore transects have a length of about 500 m and a width of 5 m. Thus, a total area of 2500 m^2^ was investigated. The entire beach surface was visually examined for plastic debris or abandoned campfire sites. The potential new plastic waste types were identified by visual inspection and comparison with figures and descriptions from published studies and were categorized into plastiglomerate^9,10^, pyroplastic^11,12^, plasticrust^13–15^, and plastitar^16^. The collected samples were subsequently stored in Ziplock bags for further analysis. A total of 32 samples of suspected new plastics were collected.

### Basic properties

The samples were photographed using a digital camera, and their maximum length, width, and height were measured with a digital caliper. The weight of each piece was measured using a precision balance (BCE623I-1S, Sartorius Lab Instruments, Göttingen, Germany). Floating tests were performed with artificial seawater (34 ‰) prepared from table salt. Each sample's color, composition, and degree of burning (melted/charred) were recorded. The suspected source of the plastic was identified where possible. Distinguishing the source of the plastic is possible when some parts of the matrix were not fully melted but still retained their original color and shape^15^. The samples were further classified into four categories: plastiglomerate^10^, pyroplastic^10,11^, plasticrust^10,13,15^, and plastitar^16^ according to the criteria outlined in previous studies^10,11,13,15,16^ and stated in the introduction.

### Infrared spectroscopy analysis

The dominating polymer type in each sample (*n* = 25) has been identified using Fourier-transform infrared (FTIR) spectroscopy^35^. A small plastic chip was cut out of the sample using a scalpel. The plastic polymer was examined via attenuated total reflectance ATR-FTIR spectroscopy (Vertex 80v, Bruker Optics GmbH, Ettlingen, Germany) equipped with a Platinum A225/Q diamond ATR unit (Bruker Optics GmbH, Ettlingen, Germany). Using OPUS 7.5 software (Bruker Optics GmbH, Ettlingen, Germany), the spectra were recorded in the 400–4000 cm^−1^ wavenumber range by co-adding 32 interferometer scans. The spectral resolution was set to 4 cm^−1^, and no ATR spectral intensity correction was applied to ensure comparability with the plastic spectra database. Each measurement was performed in triplicate, and the averaged spectrum was used for further analysis. More precisely, the same chip has been re-analyzed three times, with a new background scan performed between each measurement. Good signal-to-noise ratios with typical peak absorbances in the order of 0.1 have been observed, resulting in an excellent reproducibility of the spectra. Open Specy open-source software and its onboard spectral library^36,37^ were used to further pre-process the spectra and polymer identification. In particular, a thresholding iterative polynomial baseline correction method has been applied with the order of the polynomial chosen to mostly remove the baseline drift and broad features in the spectrum. Spectral regions identified to show no or below-threshold signals are set to zero absorbance. This way, a higher weight was put on narrow-bandwidth vibrational signatures characteristic of the different plastic polymers. Moreover, sample and instrument-specific signal intensity differences between sample and reference spectra have been removed by using absorbance intensities instead of absorbance, i.e., all spectra have been normalized with respect to the absorbance of the highest peak in the spectrum. The spectral assignment was based on Pearson’s r correlation between sample and reference spectra. The reliability of the assignment has been checked by analyzing measured ATR spectra of plastic samples of commercial PE, PP, and PET. For these validation samples, the Pearson correlation coefficients were in the ranges 0.85–0.99. Therefore, only matches with a Pearson correlation coefficient ≥ 0.85 were accepted for the polymer characterization of all samples.

### Organic geochemistry

Six samples of plasticrust (*n* = 3), plastiglomerate (*n* = 2), and plastitar (*n* = 1) were further examined for associated chemical contaminants. The outsides of all samples were cleaned by repetitive washing with hot, deionized water and finally with a solvent mixture of *n-*hexane/isopropanol (9/1: v/v). After drying, an aliquot was crushed and ground in a mortar and extracted by ultrasonication with dichloromethane (DCM), followed by methanol/DCM (9/1: v/v) to utilize the swelling effect of methanol and finally with DCM. Extracts were combined and dewatered over NaSO_4_ before being taken to dryness. An aliquot of the total extract was redissolved in hexane/DCM (9/1: v/v) and sorbed to silica gel which was applied onto an SPE cartridge for compound class separation into aliphatic and aromatic hydrocarbons and polar components employing an LC-TECH automated SPE system. Separation afforded an 8 ml SPE column (2.8 g Silica 60 mesh, 25–40 μm) using solvents of increasing polarity. Aliphatic hydrocarbons were eluted with n-hexane, aromatic hydrocarbons were eluted using a mixture of n-hexane and DCM (3:2, v/v), and NSO compounds were eluted with DCM:MeOH (1:1, v/v). Aromatic and polar NSO-fractions were spiked with deuterated pyrene and analyzed by GC/MS using an Agilent 5977MS interfaced to an Agilent 7890 gas chromatograph (GC) equipped with a DB-5MS capillary column (Agilent DB5-MS; 30 m length, 0.25 mm inner diameter, 0.25 μm film thickness). The gas chromatograph oven temperature program was: 70 °C (5 min isothermal) to 140 °C at 10 °C/min, then to 325 °C at 3 °C/min (held isothermally for 7 min). The quadrupole mass spectrometer was operated at an ionization energy of 70 eV in scan mode with 1.0 scans per second in the m/z 50–750 mass range. Compounds were identified based on their mass spectra via comparison with authentic standard mixtures and literature data.

## Results

Thirty-two samples were collected on the coastal transect. Seven samples were excluded from further analysis because they did not contain plastic. Specifically, six samples were coral fragments partially covered with tar, and one was a coral fragment covered with algae. Thus, 25 samples containing plastic were identified and selected for further analysis (Table 1).

**Table 1:** List of sample types and their basic properties.

No	Sample ID	Type	Description	Weight (g)	dimension (mm)	Likely source of plastic	Polymer identified
1	PJY1-01	Plastiglomerate	Coral fragments and wood in a blue/black (burned) plastic matrix	110.5	153 × 102 × 30	Ropes	HDPE
2	PJY1-02	Plasticrust	Blue and black (burned) plastic encrusting a coral fragment	22.1	85 × 47 × 11	Unclear	LDPE
3	PJY1-03	Pyroplastic	Partially melted pink plastic	8.2	48.6 × 36.6 × 26	Unclear	PP
4	PJY1-04	Plasticrust	Black (burned)/blue plastic encrusting a coral fragment	38	73 × 45 × 20	Unclear	LDPE
5	PJY1-05	Plasticrust	Black (burned)/blue plastic encrusting a coral fragment	14.7	37 × 30.5 × 19	Unclear	LDPE
6	PJY1-06	Plasticrust	Light green melted plastic encrusting wood and a coral fragment	16	46 × 29 × 21	Unclear	HDPE
7	PJY1-08	Plastiglomerate	Coral fragment in a light blue melted plastic matrix	7	35 × 22 × 14	Unclear	PU
8	PJY1-10	Plasticrust	Blue plastic fragment inside a coral corallite	29	59 × 34 × 15	Unclear	HDPE
9	PJY1-11	Plasticrust	Green melted plastic encrusting a coral fragment	15.6	46 × 38 × 19	Unclear	PP
10	PJY1-12	Plastiglomerate	Green melted and black (burned) plastic encrusting corals fragments	50.5	69 × 66 × 27	Unclear	PU
11	PJY1-13	Plasticrust	Blue partially melted plastic and white rope encrusting a coral fragment	4.6	25 × 19 × 13	Ropes	PP
12	PJY1-14	Plasticrust	Black (burned) and dark green melted plastic encrusting a coral fragment	7.9	34 × 20 × 18	Ropes	HDPE
13	PJY1-15	plastiglomerate	Coral fragments and wood in yellow-brownish melted plastic	10.3	48 × 29 × 17	Unclear	SAN
14	PJY1-16	Plastiglomerate	Coral fragments and sand in a dark red plastic matrix	15.3	68 × 49 × 16	Unclear	HDPE
15	PJY1-17	Plasticrust	Blue melted plastic encrusting coral fragments	9.4	34 × 24 × 24	Unclear	PP
16	PJY1-20	Pyroplastic	Melted green plastic film	3.1	43 × 34 × 8	Packaging material	PP
17	PJY1-21	Plastitar	Plastic cap, coral fragment, mollusk fragment, leave, sand in tar matrix	12.4	49 × 41 × 24	Cap/tube	LDPE
18	PJY1-22	Plasticrust	Blue melted plastic and black burned plastic encrusting coral fragments	18.7	51 × 30 × 19	Unclear	LDPE
19	PJY1-23	Plasticrust	Green melted plastic encrusting a coral fragment	7	34 × 18 × 12	Unclear	PP
20	PJY1-25	Plastiglomerate	Green partially melted plastic bounding coral rubble	2.5	35 × 13 × 7	Coarse fiber/rope	PP
21	PJY1-27	Plasticrust	Blue melted plastic encrusting coral fragments	14.4	40 × 25 × 16	Unclear	LDPE
22	PJY1-28	Plasticrust	Greenish-white plastic matrix encrusting a wood fragment	1.8	17 × 14 × 7	Unclear	HDPE
23	PJY1-30	Plasticrust	Green/black (burned) plastic encrusting a coral fragment	3.1	21 × 15 × 9	Unclear	PP
24	PJY1-31	Pyroplastic	Partially melted white plastic	0.5	16 × 8 × 8	Packaging material	HDPE
25	PJY1-32	Plastiglomerate	Blue plastic attached to serpulid worm tubes	0.9	41 × 23 × 8	Coarse plastic fabric	PP

*HDPE* high-density polyethylene, *LDPE* low-density polyethylene, *PP* polypropylene, *PU* acrylates/polyurethane/varnish, *SAN* Styrene acrylonitrile.

**Figure 2:**
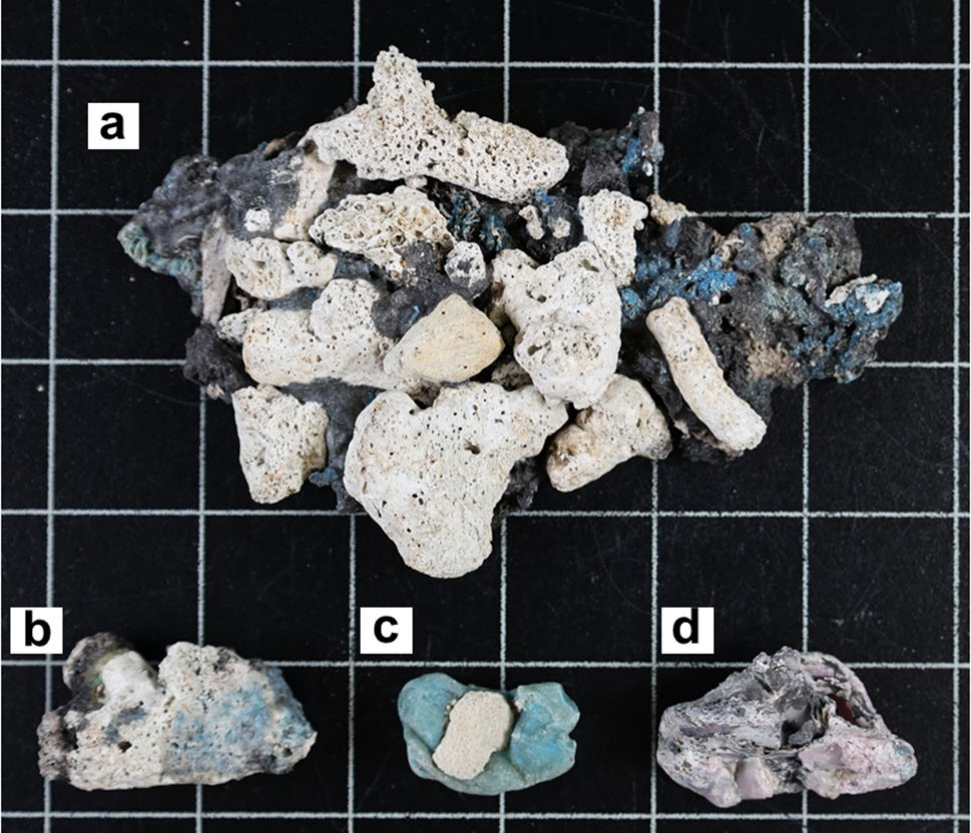
Different plastic pollution types with variable proportions of plastic. (**a**) Plastiglomerate formed by coral fragments, wood, and a matrix of melted (blue) and partly charred (black) plastic (PJY1-01). (**b**) Melted plastic encrusting the surface of a coral fragment and filling its corallites, defined as a clastic plasticrust (PJY1-22). (**c**) Melted plastic matrix with a single rubble-sized coral fragment and carbonate sand, defined as plastiglomerate (PJY1-08). (**d**) Pyroplastic of faded pink color and low sphericity (PJY1-03). Plasticrust (**b**), plastiglomerate (**a**,**c**), and pyroplastic (**d**) represent a continuum with increasing plastic content. Plasticrusts (**b**) and pyroplastic (**d**) can be formed by fragmentation of plastiglomerate.

All samples differed in mass and dimensions but were roughly similar in angularity and degree of plastic melting. Coral fragments are the most important natural component in all samples (Fig. 2). The plastiglomerate samples collected (*n* = 7) were all of the clastic type^10^, defined as a mixture of natural and artificial material in a molten plastic matrix of various colors. The plastic matrix of several plastiglomerate and plasticrust samples was burned more intensively and partially showed a black charcoal color. Plasticrust (*n* = 14) was found exclusively in its clastic form. In all samples except one, the melted plastic partially filled the corallites of the coral skeleton. In one sample, a piece of plastic debris was stuck in a coral corallite. Only a few fragments of pyroplastic (*n* = 3), melted or burned plastic that has lost its original shape and color^11^, were present. All pyroplastic samples were in the “early stage of formation,” meaning that the plastic had been recently burned and retained some of its color and original shape^15^. Several samples were found on the backshore within fire pits used for unregulated waste burning.

A sample consisting of tar-bonded plastic and natural debris was identified as plastitar (*n* = 1). The sample resembles a tar ball^38^ but with the addition of extraneous material. In contrast to the original description^16^, the plastitar was not attached to a rock outcrop but occurred in a clastic variety. However, only the pyroplastic (*n* = 3) and not the plastitar sample were buoyant during the flotation test.

**Figure 3:**
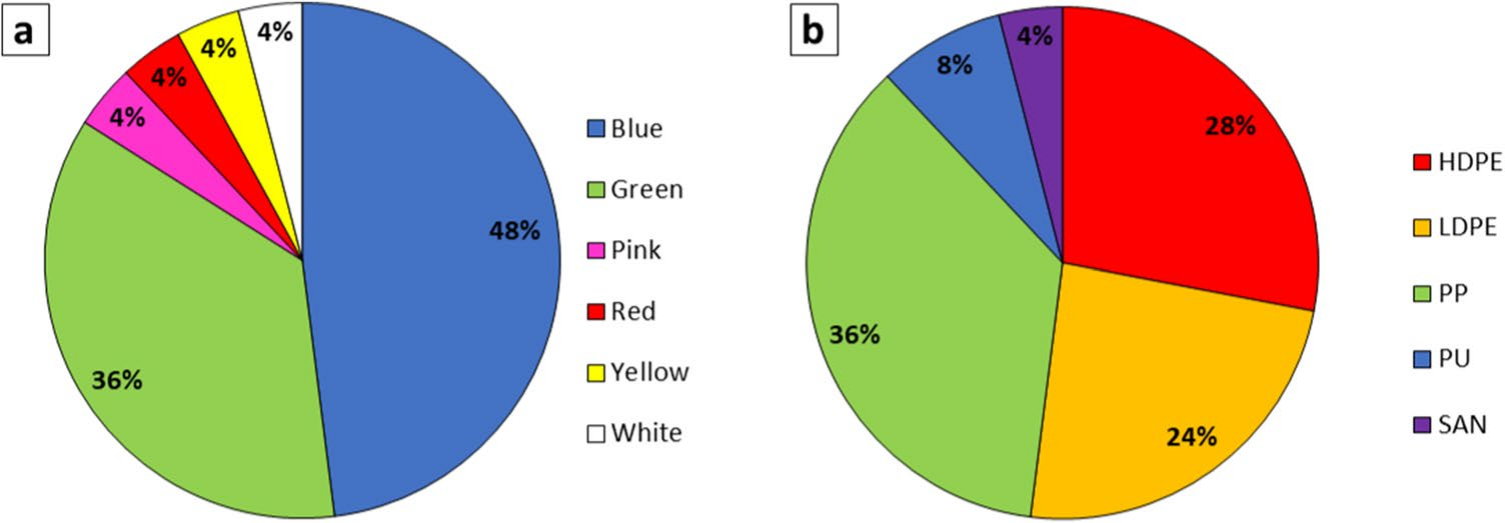
The abundance of different original colors (**a**) and polymer types (**b**) of the plastic samples collected at the northern coastline of Panjang Island, Banten Bay.

Most of the molten plastic retained its original color. The most common color observed was blue (48%), followed by green (36%), pink (4%), red (4%), yellow (4%), and white (4%) (Fig. 3). The source material of the plastic that could be identified in this study includes ropes, a cap/tube, packaging material, coarse fibers, and coarse plastic fabric (Table 1).

**Figure 4:**
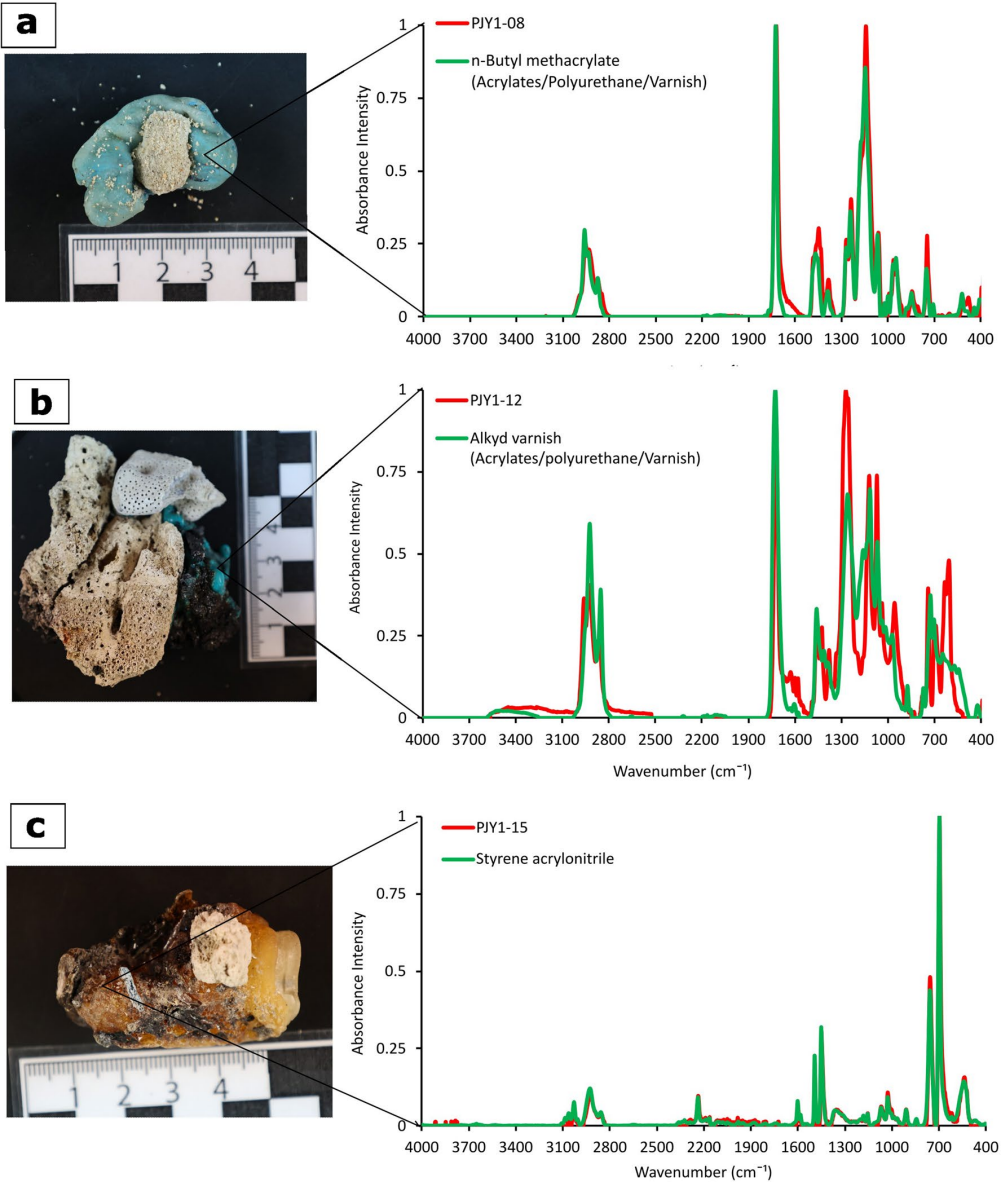
Examples of plastiglomerates with an unusual plastic matrix. (**a**) The FTIR spectrum of the plastic matrix of sample PJY1-08 shows a match of r = 0.98 with a reference spectrum of Acrylates/Polyurethane/Varnish. (**b**) The FTIR spectrum of the molten plastic matrix of sample PJY1-12 shows an agreement of r = 0.87 with an Acrylate/Polyurethane/Varnish reference spectrum. (**c**) The FTIR spectrum of sample PJY1-15 confirms the presence of styrene-acrylonitrile copolymer with an agreement of r = 0.98.

**Figure 5:**
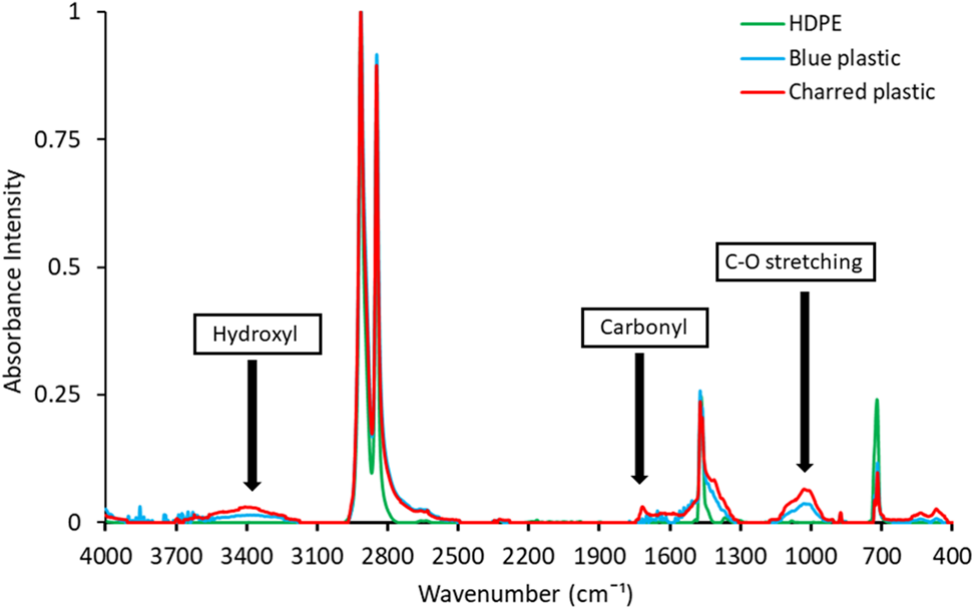
Effects of oxidative weathering on the FTIR-Spectrum of an HDPE sample (PJY1-01). Compared to the reference spectrum, the molten (blue) and charred parts of the matrix show additional absorbance bands in the 1000–1200 cm^−1^, 1700–1800 cm^−1^, and 3030–3675 cm^−1^ ranges. These can be attributed to hydroxyl groups, carbonyl groups, and C–O stretching resulting from oxidation. The carbonyl-associated band is only present in the charred part of this sample. The hydroxyl group and C–O stretching bands show higher intensities in the charred region compared to the molten but uncharred part of the sample.

**Figure 6:**
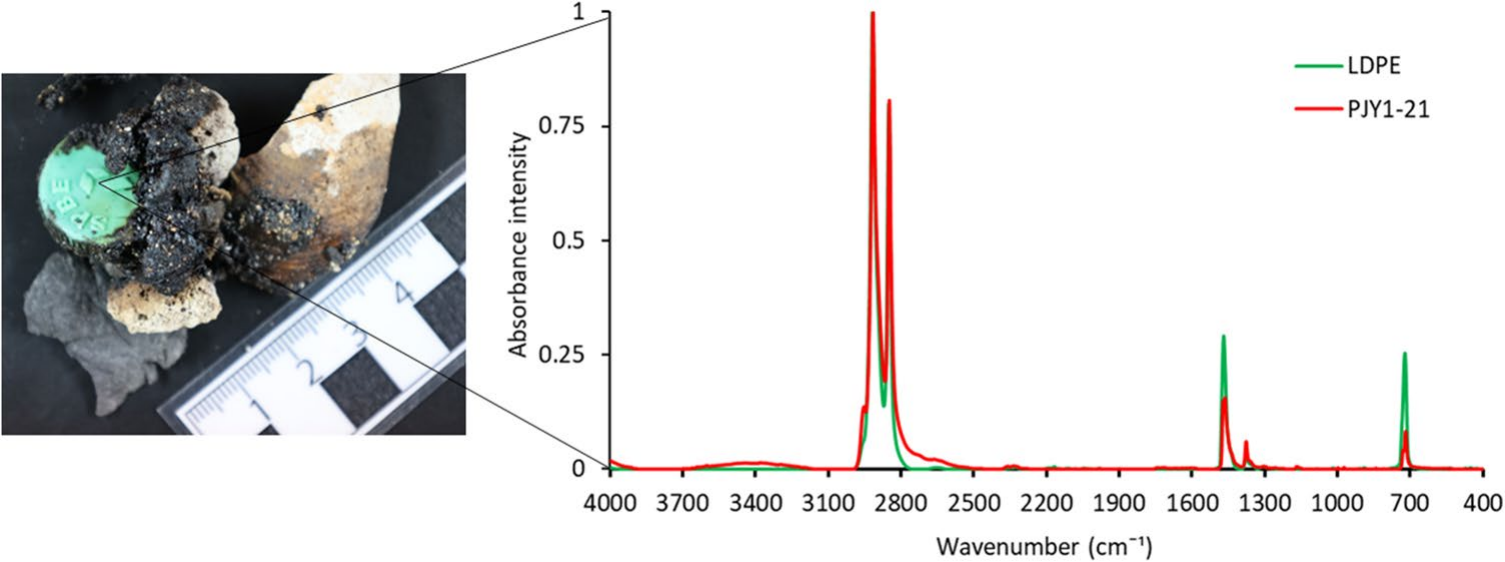
Plastitar (PJY1-21) consisting of plastic debris (lid) and natural material (coral and shell fragments) in a tar matrix. The plastic fragment was identified as LDPE using FTIR.

ATR-FTIR spectroscopy (Figs. 4, 5, and 6) confirmed polyethylene (PE, *n* = 13), polypropylene (PP, *n* = 9), acrylates/polyurethane/varnish (PU, *n* = 2; a class of difficult to distinguish polymers as defined by Primpke et al.^35^ based on a hierarchical cluster analysis of a large dataset of microplastic samples), and a copolymer of styrene and acrylonitrile (SAN, *n* = 1) as polymers (Fig. 2). The polyethylene was further differentiated into low-density polyethylene (LDPE) and high-density polyethylene (HDPE) based on the presence or absence of a 1377 cm^−1^ band in the spectra^39^. In total, 28% of all samples were assigned as HDPE (*n* = 7) and 24% as LDPE (*n* = 6). The differentiation between LDPE and HDPE allows a better constraint of the source material since HDPE is mainly used for rigid materials (e.g., cans and pipes), while LDPE is typically used for elastic and transparent products, such as plastic bags and films. Additional absorbance bands, not present in the library spectra of polymers, were commonly observed in the 1000–1200 cm^−1^, 1700–1800 cm^−1^, and 3030–3675 cm^−1^ ranges (Fig. 5). These bands were typically present in the melted plastic. Still, they showed a higher intensity in the charred parts of the same samples (Fig. 5).

The molten plastic matrix (PU) of a plastiglomerate sample (PJY1-12, Fig. 4) yielded exceptionally high amounts of phthalates in its uncharred portion, which were reduced to less than one-tenth in the charred part of the sample (Table 2). The high phthalate concentration in the uncharred part was accompanied by a high degree of solvent extractability (58% wt). Charring of the plastiglomerate sample reduced the solvent extractability to about 8% [wt] (Table 2). The PAH concentration is significantly increased in this sample’s black, charred portion compared to its green, melted part (Table 2, Fig. 4, sample PJY1-12). In contrast, plastiglomerate samples with a matrix of polyolefin (PE/PP, Fig. 2: PJY1-01, and PJY1-14) show no systematic increase in PAH concentrations in their charred portion (Table 2). The GC/MS analysis indicated that sample PJY1-21 (Fig. 6) is agglutinated by a tar matrix, and was therefore categorized as a plastitar. This sample also showed the highest PAH concentration (Fig. 2).

**Table 2:** Organic contaminants associated with plastic pollution.

Sample	Plastic pollution type	Description	Weight specimen [mg]	Extract [mg]	ppm extract [µg/g]	PAH [µg/g specimen] aromatics	Phthalates [µg/g specimen] aromatics	Phthalates [µg/g specimen] polars	Phthalates [% of polars]
PJY1-01	Plastiglomerate	blue plastic (HDPE)	93.1	1.8	19,334	0.8	0.2	3.9	0
PJY1-01		charred plastic particle	119.0	16.8	141,176	0.2	0.3	9.7	0
PJY1-11	Plasticrust	green plastic (PP)	51.7	6.3	121,857	1.0	0.4	417	0
PJY1-12	Plastiglomerate	green plastic (acrylates/PU/varnish)	36.1	21.0	581,717	1.1	1.1	369,769	64
PJY1-12		charred plastic particle	279.9	23.6	84,316	16.9	0.2	40,976	49
PJY1-14	Plasticrust	green plastic (HDPE)	13.6	1.1	80,882	2.3	1.3	41	0
PJY1-14		charred plastic particle	34.5	1.0	28,986	0.3	1.0	23	0
PJY1-21	Plastitar	tar matrix	280.9	257.5	916,696	44.0	0.9	142	0
PJY1-30	Plasticrust	charred plastic particle (PP)	59.6	53.8	902,685	5.2	1.5	89	0

## Discussion

### Classification and origin of the new types of plastic pollution

Several new terms were introduced to classify the types of plastic pollution studied^9–13,16,18^. However, the distinction between plastiglomerate and its variants is sometimes subtle or imprecise. For example, round, molten plastic debris can be referred to as pyroplastic, while similar but more angular plastic with more extraneous materials may be better described as plastiglomerate^11^. A link between plastiglomerate and pyroplastic was observed on a pebble beach, indicating that pyroplastic can form when a plastiglomerate has lost its clasts^40^. Similarly, we observed that larger plastiglomerates on the coral-rubble-dominated beach in the Java Sea could break down into smaller fragments due to weathering or wave action, forming plasticrusts, smaller plastiglomerate, and pyroplastic (Fig. 2). These terms, therefore, describe a continuous spectrum of plastic pollution types with an increasing proportion of plastic matrix (Fig. 2). For example, the term plastiglomerate might apply if a single rubble-sized coral fragment and a few sand grains are attached to a relatively large piece of molten plastic (Fig. 2C). However, the term clastic plasticrust might be more appropriate, if the molten plastic forms only a relatively thin veneer on a similar coral clast or fills its corallites.

The original form of five samples of melted plastic debris (24%) was well enough preserved to identify their parent material (Table 1). Four pieces were melted ropes, fibers, or fabric, and one was packaging material. In addition, the plastitar sample contained a plastic cap. The original color of the plastic was at least partially preserved in all samples. The relatively well-preserved shapes and colors indicate that the samples are in their “early and middle stages of formation”^15^. This is consistent with the fact that several pieces, including pyroplastic, were recovered from fire pits on the backshore area of the beach (see Supplementary 2). These findings imply that the plastics were recently burned in situ and not transported from elsewhere. This contrasts with previous reports^11,15^, in which pyroplastic samples had a more natural rock-like shape, neutral color, and smooth surface, and were typically found in the intertidal zone.

Therefore, except for the plastitar sample, the new types of plastic pollution described in this study are interpreted to result from unregulated, on-site burning of waste. In fact, the studied coastline in the Java Sea has an order of magnitude higher concentrations of new types of plastic pollution (1 × 10^−2^ pieces per m^2^) compared to other sites, e.g., Peru (1 × 10^−3^–9.74 × 10^−5^ pieces per m^2^)^15^ and Portugal (4 × 10^−3^ pieces per m^2^)^40^. This suggests that even remote beaches of the maritime continent of Indonesia can be highly polluted by plastiglomerate and its variants.

### New polymer types in plastiglomerate

ATR-FTIR spectroscopy confirmed that most samples were composed of PE (52%) and PP (36%, Fig. 3). PE and PP are the most common polymers in plastic consumer goods, such as plastic packaging, fishing lines, containers, etc. This is consistent with the observations that the identifiable plastic items such as ropes, fibers, fabric, and packaging materials were either PE or PP (Table 1).

Previous studies on plastiglomerate and its variants have mentioned PE, PP, and PET as polymers^11,13,15,17,18,40^. This study reports acrylates/polyurethane/varnish and a styrene-acrylonitrile copolymer as additional polymer type in plastiglomerates (Table 1, Fig. 4). The IR-spectrum of sample PJY1-08 (Fig. 4a) yields the best match to the reference spectrum of polymeric *n*-butyl methacrylate (*r* = 0.98) and the IR-spectrum of sample PJY1-12 (Fig. 4b) to polyester-based alkylated varnish (*r* = 0.87), both belonging to the class of acrylates/polyurethane/varnish as defined by Primpke et al^35^. The IR-spectrum of sample PJY1-15 (Fig. 4c) can be assigned to styrene acrylonitrile (*r* = 0.98), belonging to the class of polystyrenes. Acrylates are a common ingredient in boat paints because they are known to improve water resistance^41^. Chipped boat paint is commonly found on beaches and is considered one of the most significant contributors to microplastic pollution in marine habitats worldwide^20,42–44^. Styrene-acrylonitrile copolymer (SAN) is a less commonly used plastic polymer, a tough material known for its heat resistance. The study shows that plastiglomerates and their variants can comprise more polymers than previously known.

### Weathering of plastiglomerate and its variants

The unregulated burning of plastic debris in open fire pits caused very variable degrees of melting and charring, even within a single plastic item (e.g., sample PJY1-01, Fig. 2). This was used to assess the effect of burning on plastic weathering. The FTIR spectrum of the blue molten plastic matrix of sample PJY1-01, which in some parts is still recognizable as a maritime rope, is very similar to the polyethylene reference data (Fig. 5, r = 0.98). In addition, the molten plastic shows broad bands in the spectral range between 3200–3600 cm^−1^ and 1000–1200 cm^−1^. Broad absorption bands in these regions are associated with hydroxyl groups (3030–3675 cm^−1^) and are commonly observed in weathered PE^45–47^. An increase in absorption in the 1000–1200 cm^−1^ range was previously related to the stretching vibration of C–O, also likely as an effect of oxidative weathering^45^.

It was previously reported that the charring of plastic might lead to a complete obliteration of the polymer FTIR spectrum^15,19^. In contrast, the samples from Panjang show the typical FTIR spectra of polymers even in the black, charred parts of the samples (e.g., Fig. 5). However, the intensities of the two bands at 3200–3600 cm^−1^ and 1000–1200 cm^−1^ are higher in the charred part than in the blue part of the sample, indicating more intense oxidation. The FTIR spectrum of the charred, black matrix exhibits an additional peak at 1717 cm^−1^, which was not present in the blue, molten part of the sample (Fig. 5). Peaks in the 1700–1800 cm^−1^ region typically indicate the presence of oxidation products corresponding to the vibration of carbonyl groups, hence may suggest that the polymer has been oxidized^48,49^. The carbonyl index, which measures the growth in the carbonyl (C=O) absorption relative to a stable reference absorbance band, is commonly used to assess the aging behavior of polymers^46,47,50,51^. Thus, this peak’s presence confirms that the charred is more oxidized and weathered than the only melted portion of the sample, which has retained its original color. In nature, this weathering is typically attributed to the effect of solar photo-oxidation, i.e., the degradation of polymer chains due to the combined effect of UV light and oxygen^47^. UV radiation causes the scission of C–H bonds in the polymers and the production of free radicals resulting in the formation of oxidative-functinoal groups such as carboxylic acid and hydroxyl^47^. However, photo-oxidation is generally limited to the surface layer (100 µm) of polymers^47^. In contrast, we observed the same spectral features indicative of oxidation in subsamples taken from the interior of the molten plastic matrix. This suggests that the effects of oxidation extend well below this surface layer and, therefore, cannot be solely explained by solar photodegradation. The alternative, thermal degradation of plastic, is often considered unlikely in the environments due to the high temperatures required to start thermo-oxidative reactions^47^. However, campfire temperatures would be high enough to explain thermal degradation. In general, the mechanism of thermal degradation is comparable to that of photodegradation in oxidizing environments. However, the higher temperatures result in faster reaction rates and larger amounts of free radicals available for chain scission^52^.

Consequently, plastiglomerate and its variants show a different weathering pattern than other plastic debris. Thermodegradation affects the entire plastic matrix of plastiglomerates, while solar photo-oxidation is dominant on the surface of other plastic waste in the environment. This directly impacts fragmentation behavior, as charred plastic is generally brittle and friable and has been repeatedly cited as a major source of microplastics^11,15,17,19,53^. To what degree the mechanical properties of the molten but not charred plastic are already influenced by thermo-oxidation is yet unclear and needs further investigation.

### Plastitar and persistent organic pollutants

Anthropogenic pollution in coastal regions is not limited to improper and inadequate disposal of plastic waste. There is also a risk of oil entering the marine environment through dumping or accidental spills. This is especially true in marine areas with heavy traffic of oil tankers, such as the Java Sea. Marine tar residues, weathered petroleum compounds derived from oil spills, are found in varying amounts worldwide^38^.

Plastitar has only been described once before from the Canary Islands^16^. It is a compound of tar, plastic, and natural components that adhere to and cover coastal rock outcrops^16^. We found one sample composed of a plastic cap and natural materials (coral fragments, a shell fragment, leaves, and sand) agglutinated in a tar matrix (Fig. 6). Its composition is therefore very similar to the plastitar described by Domínguez-Hernández et al.^16^, but it is not adhered to a rock outcrop and thus easier transported in the swash zone of the beach. In analogy to the distinction between “in-situ” and “clastic” plastiglomerate^9^, our sample could be classified as clastic plastitar. ATR-FTIR confirmed that the plastic fragment in the plastitar was LDPE. A previous study showed that PE and PP are common in plastitar^16^ due to their ubiquity in the marine environment^1^.

Concerns have been raised that persistent organic pollutants such as PAHs present in tar may transfer toxic, mutagenic, or hormone-like endocrine disruptors to marine organisms^16^. Currently, there are very few studies on the toxicity of marine tar residues^38^. In this study, we found a high PAH content in the tar matrix of the plastitar (PJY1-21, Fig. 6, Table 2). The clastic plastitar could pose a high risk to the marine environment because it is more mobile than the plastitar that is adhered to rock outcrops. It can be transported by waves and currents in the swash zone, even though the clastic plastitar in this study is not buoyant. The combination of toxicity in tar and plastics with unknown ecological consequences is a further major concern^16^.

The concentration of contaminants associated with plastiglomerate and its variants in this study is very high compared to other sites where charred plastic was investigated, e.g., the Maldives^19^. The overall high PAH levels in all plastiglomerate/-crust samples could possibly be related to the adsorption of PAHs onto plastic from the tar-influenced coast. However, the highest PAH concentrations of > 5 µg/g were measured in two charred samples (Table 2), one from a plasticrust of PP (PJY1-30) and one from the acrylates/PU/varnish matrix of a plastiglomerate (PJY1-12, Fig. 4). The PAH concentrations in the charred portion of sample PJY1-12 were significantly higher compared to the melted but uncharred portion. It is therefore likely that the burning of plastic in fire pits is associated with an increase in PAHs compared to other forms of plastic debris.

One plastiglomerate sample with an acrylates/PU/varnish matrix (PJY1-12, Fig. 4) yielded exceptionally high amounts of phthalates. Phthalates are used as plasticizers, i.e., chemicals that are added to modify the properties of plastic to make them softer or more durable. Even at low concentrations phthalates can act as endocrine disruptors in marine invertebrates, such as bivalves^54^. Phthalates were detected in the sediments and waters of mangrove ecosystems and can accumulate in mangrove leaves with possible effects on plant biosynthesis and human health^55^. Their effect on corals is still understudied, but several studies now show that phthalates are a novel, but ubiquitous contaminants in reef sediments^20^, coral tissue^56–58^ and even the skeleton of reef corals^58^. Phthalates and other persistent organic pollutants are also known to negatively impact seagrass ecosystem, e.g. by the disruption of metabolic processes^24^. Phthalates present in microplastic in coastal environments^20^, could become bioavailable to marine biota if consumed. As discussed before, charred plastic might be an important source of microplastic. This suggests that the degradation of plastiglomerate and their variants could be an important vector for the transfer of persistent organic pollutants in coral reef, seagrass and mangrove environments.

## Conclusion

The remote northern beach of Panjang Island in the Java Sea shows the worldwide highest concentration of plastiglomerates and its variants described so far. The most common plastic pollution type are plasticrust, plastiglomerate, and pyroplastic. These items appear to originate from the uncontrolled burning of plastic debris on-site at the beach. Pyroplastics and many plasticrusts likely originate from the decomposition of larger plastiglomerates and form a continuous spectrum of plastic pollution types with an increasing proportion of partially melted plastic. The dominant natural component in these new types of plastic pollution is coral rubble from the close-by reef system.

Due to their ubiquity in the environment, polyolefins (PP/PE) are the most common polymers in the samples studied here. However, acrylates/polyurethane/varnish and styrene-acrylonitrile, are two types of polymers that are described for the first time as a component of plastiglomerates. The FTIR-spectra of the polyolefins show bands in the 3200–3600 cm^−1^, 1700–1800 cm^−1^ and 1000–1200 cm^−1^ region, which are interpreted to results from thermo-oxidation by burning. This thermo-degradation leads to brittle behaviour of the most affected charred portions of the samples, which is believed to contribute to degradation and increased microplastic formation.

One sample of clastic plastitar, which consisted of plastic debris and natural material in a tar matrix, was found in the study area. Considering the intense plastic pollution and heavy traffic of oil tankers in the Java Sea, plastitar may be more common than previously realized. The clastic plastitar could pose a high risk to the marine environment because it is more mobile than the plastitar that is adhered to rock outcrops.

Most of the tested samples in this study showed high content of toxic persistent organic pollutants. The plastitar sample yielded an exceptionally high PAH concentration, while the plastic matrix of an acrylates/polyurethane/varnish sample showed very high phthalate levels. The same plastiglomerate sample also produced high PAHs, with the charred portion’s PAH concentration significantly increasing compared to the non-charred portion. Therefore, partial burning of plastic is likely generated PAHs.

The three most iconic tropical habitats are coral reefs, seagrass meadows and mangrove forests, which provide important ecosystem services. Our study area is located close to all of these habitat types. Local burning of plastic waste leads to changes in their physical and chemical properties, such as the degree of thermo-oxidative weathering and increased PAH concentrations. This could also negatively impact adjacent ecosystems. Plastiglomerates and their variants can act as a source of microplastics and as an important vector for the transfer of toxic persistent organic pollutants (POPs) to coastal habitats. These new types of plastic pollution must therefore be considered in the environmental management of tropical coastal ecosystems such as coral reefs, seagrass meadows, and mangrove forests.

## Supplementary Information

Supplementary Tables.

Supplementary Figures.

## Data Availability

The datasets generated during the current study are available in the Supplement 1.
