# Supplementary material for: Plastiglomerates from uncontrolled burning of plastic waste on Indonesian beaches contain high contents of organic pollutants
Source: Sci Rep. 2023 Jun 27;13:10383. doi: 10.1038/s41598-023-37594-z (PMC10300024; doi:10.1038/s41598-023-37594-z)
Supplement: Supplementary file 2 — Supplementary Figures. [file 41598_2023_37594_MOESM2_ESM.pdf]

Supplement 2

Abandone campfire and plastic formation in Panjang Island

Figure Supplement 2.1. Abandon campfire and plastic formation (sample PJY1-01) on the coral rubble dominated beach of Panjang Island, Java Sea

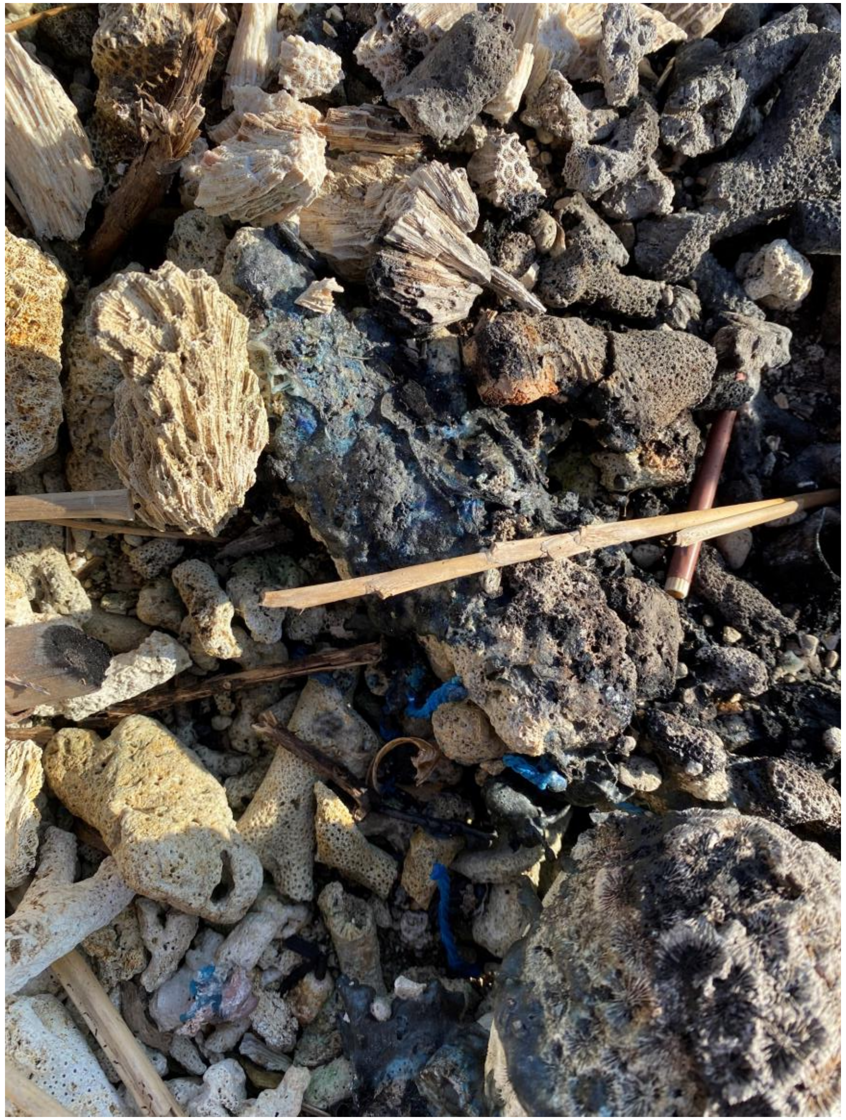

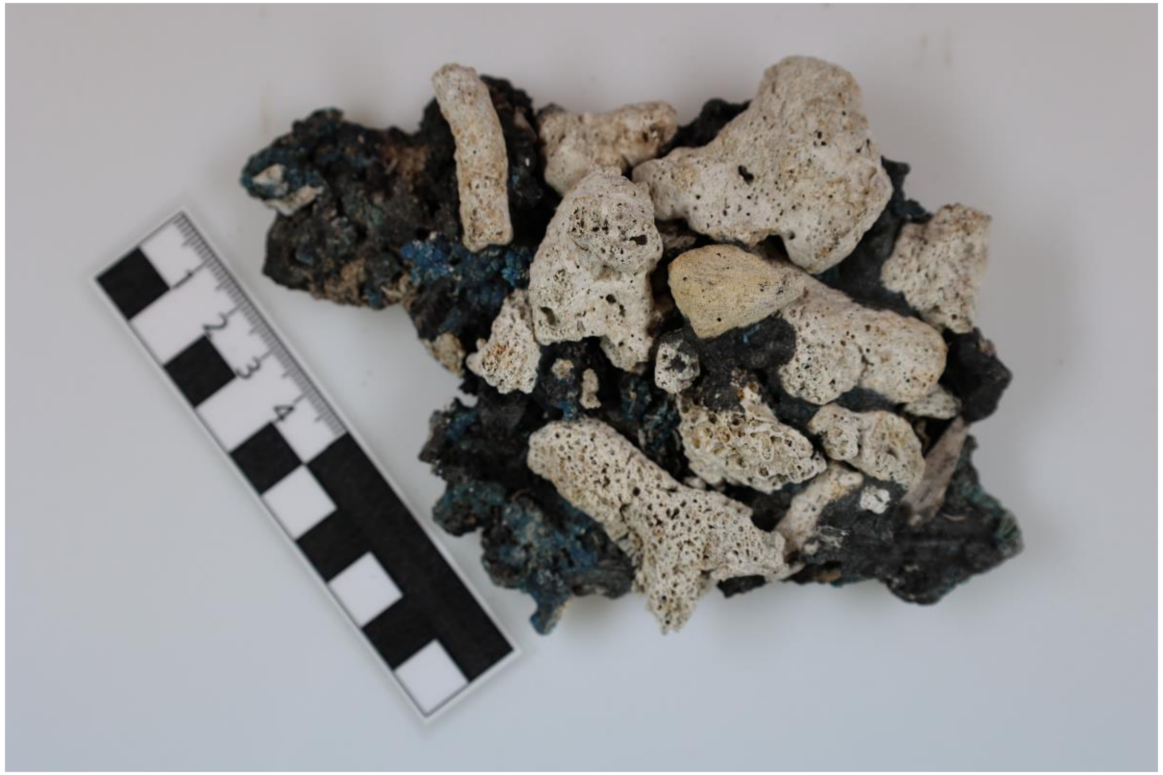

PJY1-01

Figure Supplement 2.2. Abandon campfire and plastic formation (sample PJY1-04) on the coral rubble dominated beach of Panjang Island, Java Sea

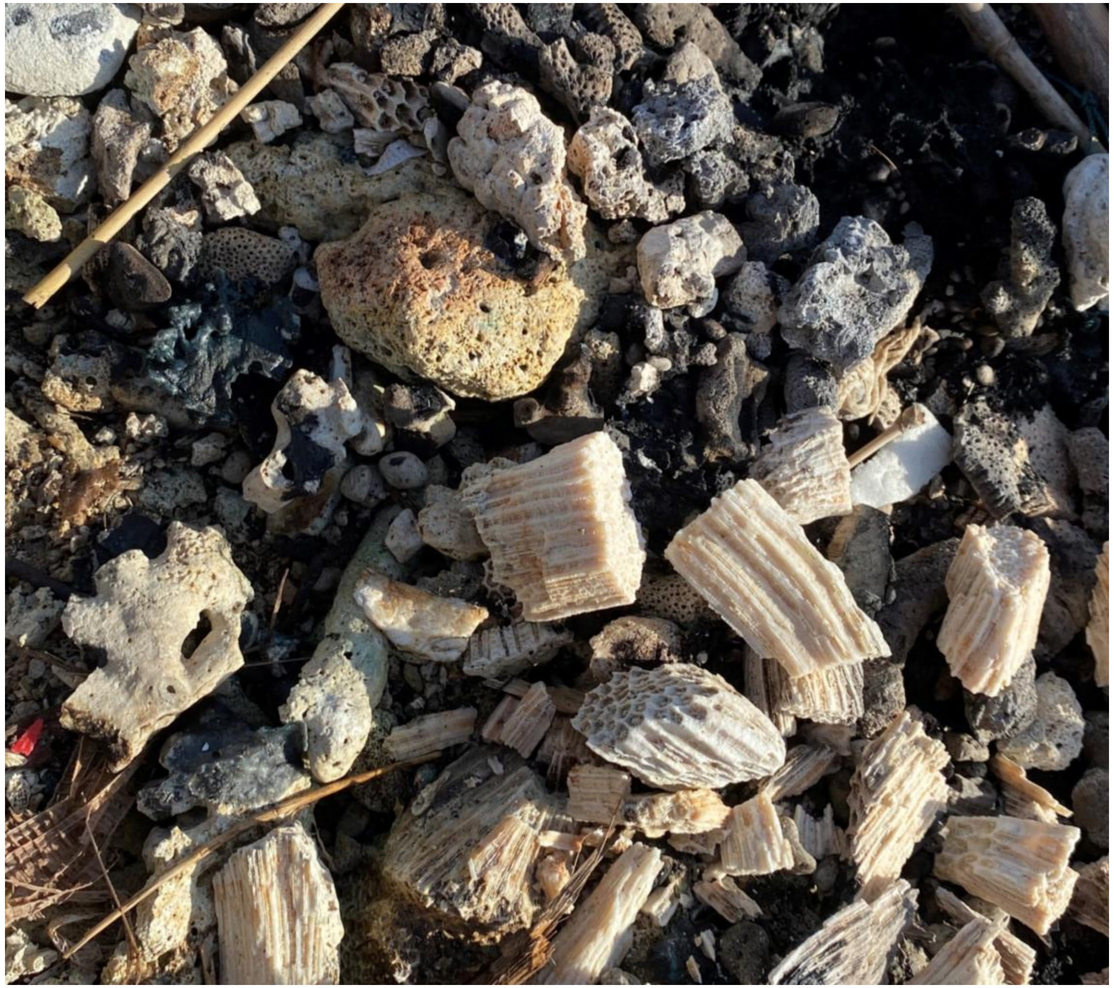

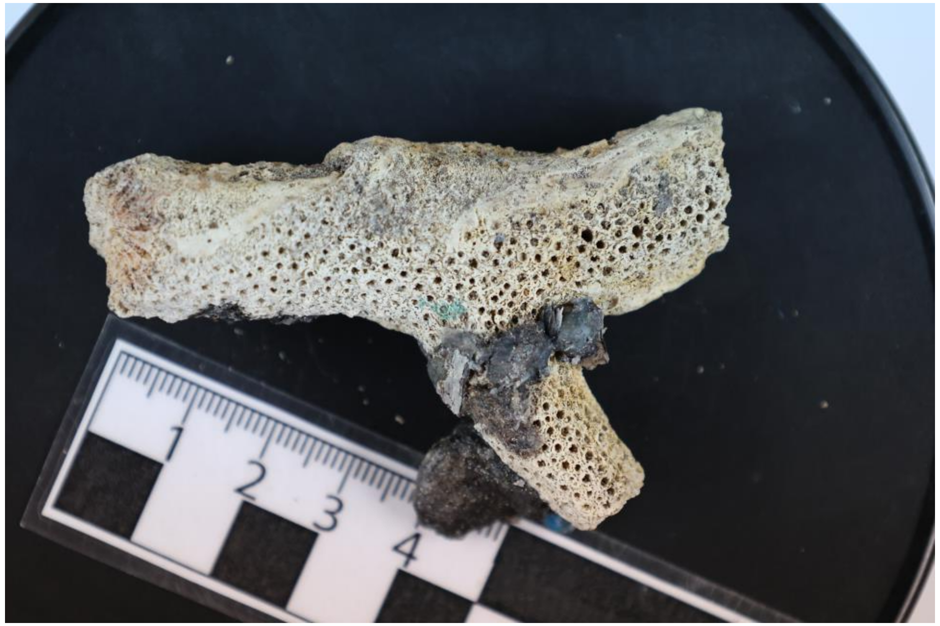

PJY1-04

Figure Supplement 2.3. Abandon campfire and plastic formation (sample PJY1-11 and PJY1-23) on the coral rubble dominated beach of Panjang Island, Java Sea

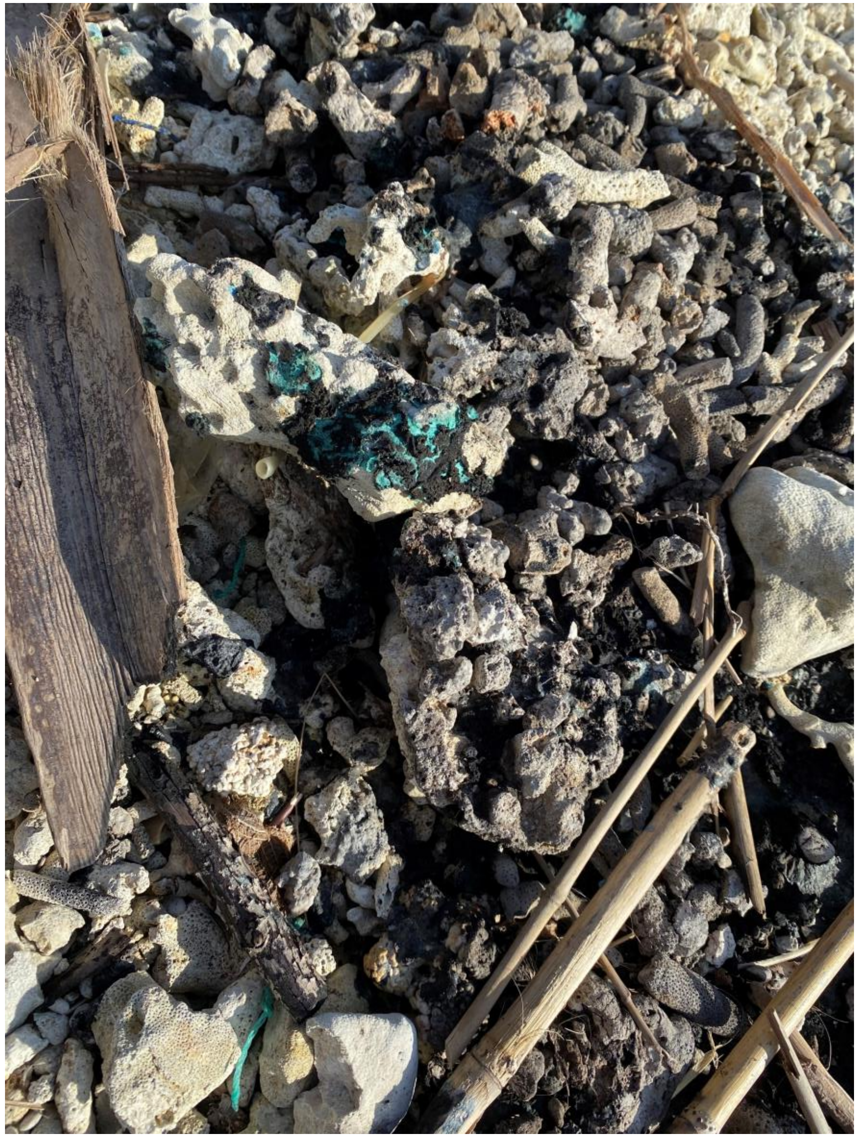

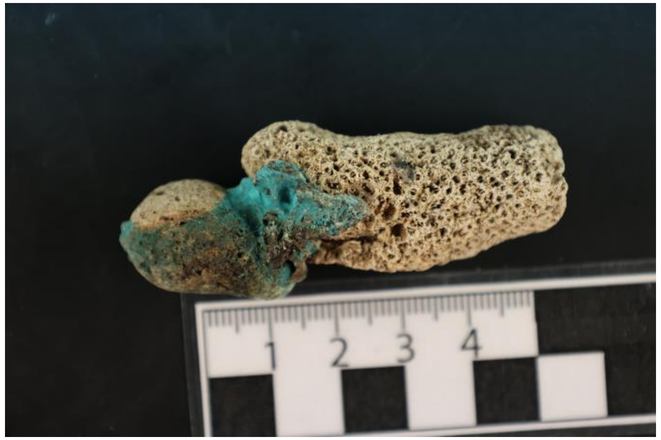

PJY1-11

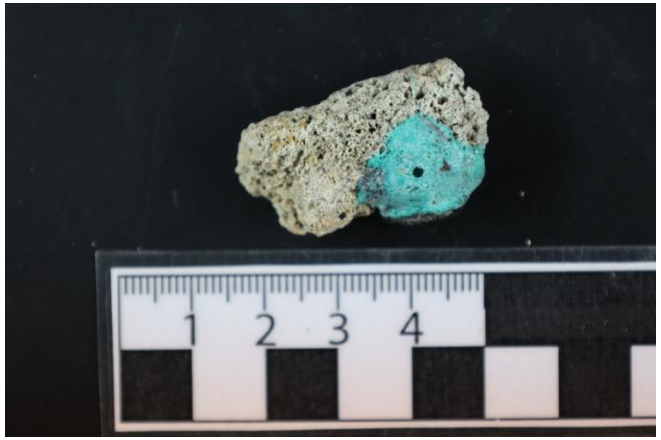

PJY1-23

Figure Supplement 2.4. Sample PJY1-12 on the coral rubble dominated beach (Panjang Island, Java Sea)

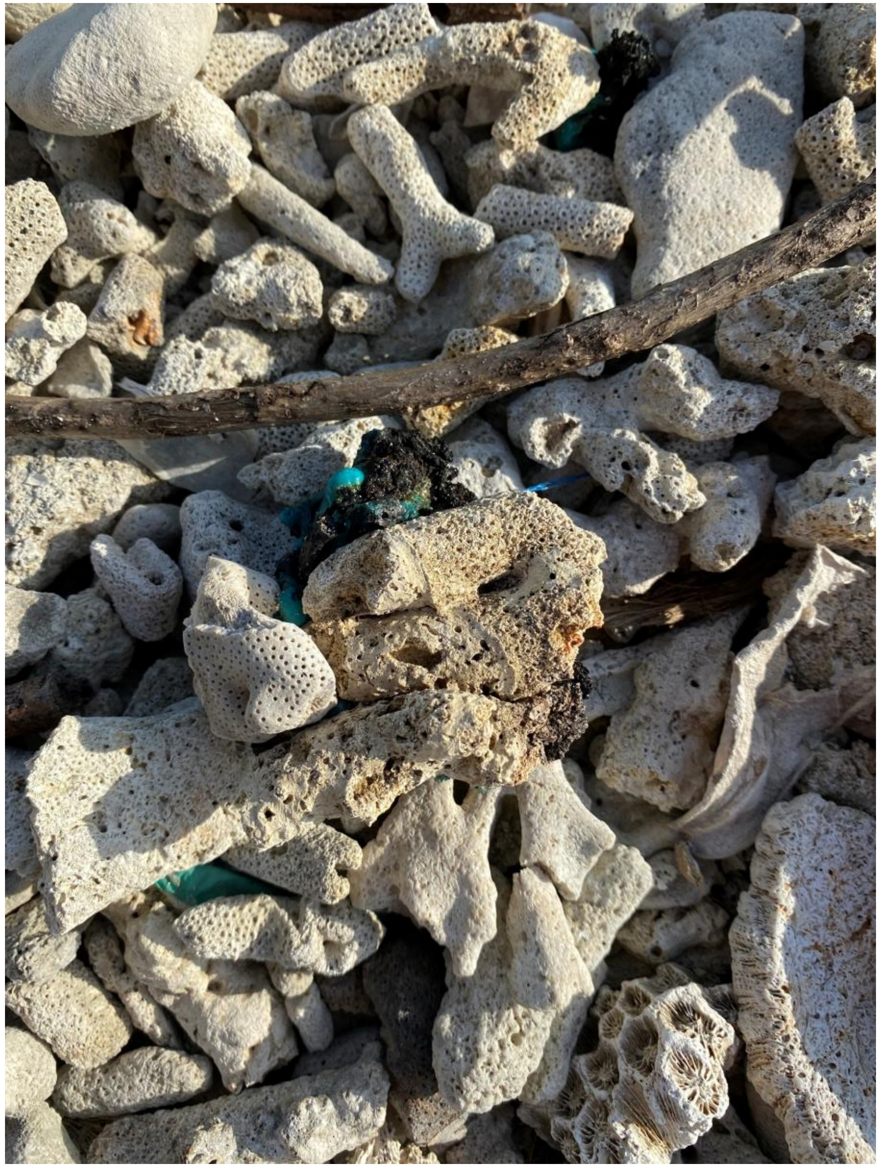

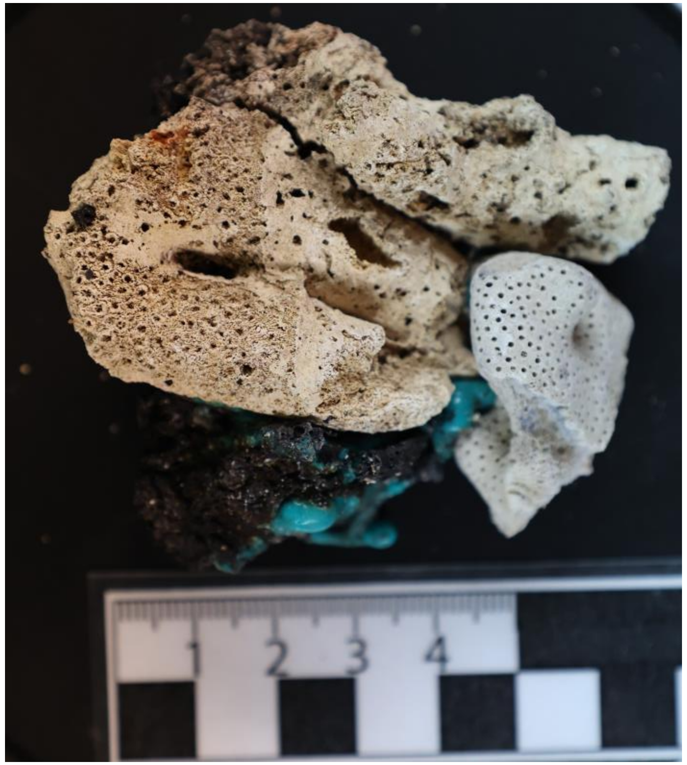

PJY1-12

Figure Supplement 2.5. Sample PJY1-20 on the coral rubble dominated beach (Panjang Island, Java Sea)

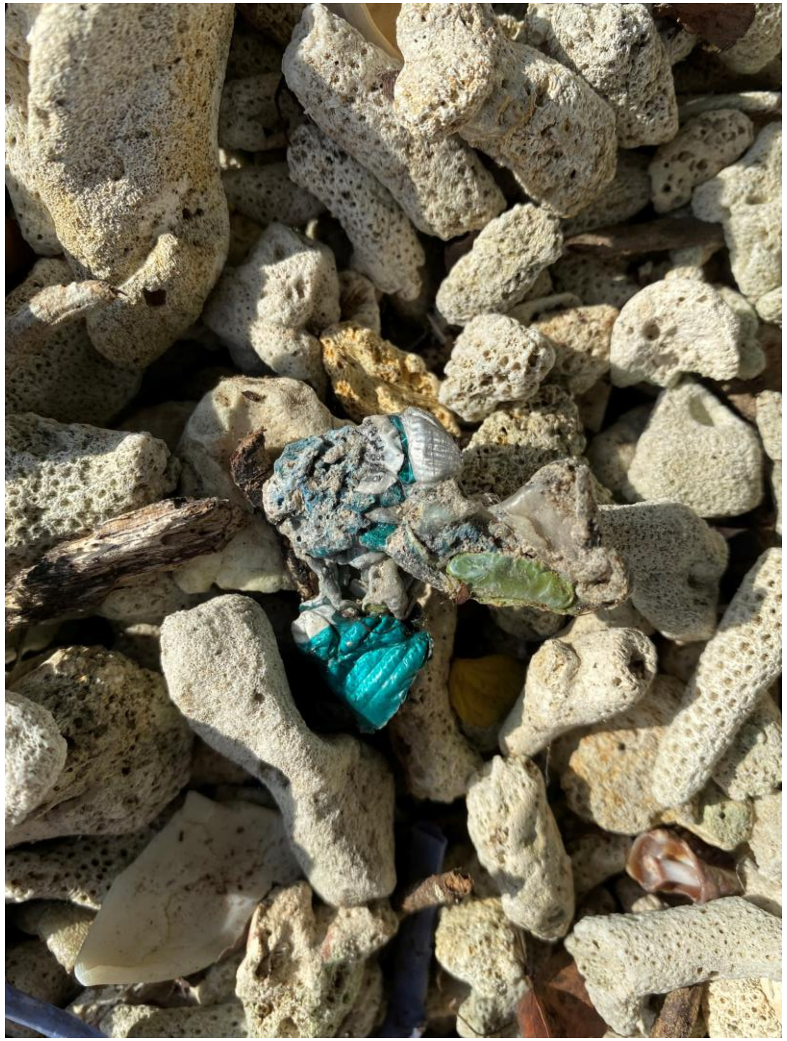

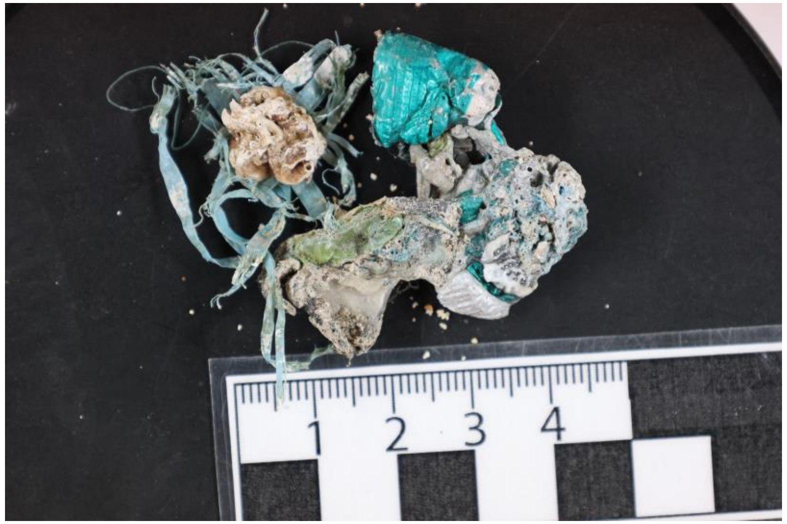

PJY1-20
